# Profiles of Environmental Mold: Indoor and Outdoor Air Sampling in a Hematology Hospital in Seoul, South Korea

**DOI:** 10.3390/ijerph15112560

**Published:** 2018-11-15

**Authors:** Sung-Yeon Cho, Jun-Pyo Myong, Won-Bok Kim, Chulmin Park, Sung Jeon Lee, Sang Hyeon Lee, Dong-Gun Lee

**Affiliations:** 1 Division of Infectious Diseases, Department of Internal Medicine, College of Medicine, The Catholic University of Korea, Seoul 16591, Korea; cho.sy@catholic.ac.kr (S.-Y.C.); symonlee@catholic.ac.kr (D.-G.L.); 2 Vaccine Bio Research Institute, The Catholic University of Korea, Seoul 06591, Korea; skaks301@naver.com (W.-B.K.); micropak@catholic.ac.kr (C.P.); 3 Catholic Hematology Hospital, Seoul St. Mary’s Hospital, Seoul 06591, Korea; 4 Department of Occupational and Environmental Medicine, College of Medicine, The Catholic University of Korea, Seoul 06591, Korea; 5 Center for Occupational and Environmental Medicine, Seoul St. Mary’s Hospital, Seoul 06591, Korea; wowolsj@hanmail.net (S.J.L.); lee-st89@naver.com (S.H.L.)

**Keywords:** air filters, environmental monitoring, fungi, hospital

## Abstract

Inhalation of fungal spores can cause various spectrums of fungal diseases in immunocompromised hosts. The aim of this study was to evaluate the concentrations and profiles of fungal species in air collected at different locations in hematology wards and outside of the hospital in Seoul St. Mary’s Hospital over the course of a year. Air sampling was performed at four locations—outside the hospital (O), in the general ward (GW), in the lounge in the cleanroom (CR_L_), and in the patients’ room in the cleanroom (CR_R_)—by using Andersen single-stage air sampler at every two weeks between May 2017 and May 2018. The results showed higher mean fungal density in summer, and the concentrations of fungi decreased as follows: O (954.8 colony-forming units, CFU/m^3^) > GW (4.2 CFU/m^3^) > CR_L_ (0.7 CFU/m^3^) > CR_R_ (0 CFU/m^3^). *Aspergillus* was most prevalent both inside (47%) and outside (62%) of the hospital. However, the outdoor fungal profile was more diverse than the indoor profile. Within the hospital, *Penicillium* was the second most dominant species. In conclusion, the outdoor fungal profile is diverse even in Seoul, a highly urbanized area in Korea. The distribution of indoor air fungi is significantly different from outdoor due to air quality systems. Heating, ventilation, and air conditioning (HVAC), as well as high-efficiency particulate air (HEPA)-filtered systems should be established to effectively reduce levels of indoor fungi.

## 1. Introduction

Indoor air quality in hospitals is an important factor for control of nosocomial respiratory infections, especially in immunocompromised hosts. A variety of microorganisms are found in environmental reservoirs such as soil, water, dust, and decaying organic matter, etc. Once such materials are brought into a healthcare facility by such possible vehicles as people, air currents, or construction material, the residing microorganisms can proliferate in various indoor ecological niches [1]. Such microorganisms, including bacteria, viruses, or fungi, can cause airborne infections. Of note, inhalation of fungal spores can lead to various spectrums of fungal diseases in susceptible hosts. As the numbers of elderly patients, cancer patients, solid organ transplantation patients, and hematopoietic stem cell transplantation (HSCT) recipients increase, the importance of environmental data on fungi is becoming more recognized [2]. Of those immunocompromised hosts, patients with hematology diseases who received intensive chemotherapy or HSCT have the highest risk of invasive fungal diseases (IFDs) [3]. Although the morbidity and mortality associated with invasive fungal diseases are still high, the available therapeutic antifungal agents are limited compared with that of antibacterial agents [4].

The density of environmental microorganisms in the air is known to be decrease in the following order: outside > lobby > general wards (GW) > intensive care units, operating room, and isolated room with specific-purposes [5,6,7,8,9,10]. According to several previous studies, fungal concentrations in specialized hospital environments (i.e., hematology unit with cleanroom) equipped with high-efficiency particulate air (HEPA) filters are usually lower (<5 colony-forming units, CFU/m^3^) than in unequipped environments [5,6,7,8]. However, moderate fungal exposure has been demonstrated even in a qualified hospital associated with hospital remodeling [9,11]. In addition, a recent study of fungal concentrations in six hospital lobbies in Korea revealed a geometric mean (GM) fungal concentration of 77 CFU/m^3^, even in an environment equipped with ‘heating, ventilation, and air conditioning (HVAC)” system [10]. Therefore, it is important to guarantee the air quality at a specific place in the hospital to control fungal infections therein.

Information on species distributions and fungal sources, as well as the performance of HVAC systems, can provide an important indication for devising the appropriate guidelines for preventive strategies against nosocomial fungal infections. Since hospital indoor fungi can originate from an outdoor environment via the airflow, more effective air quality control to reduce fungal colony counts, including HVAC systems, might be required. On the other hand, an infection control team should identify other potential fungal sources, or the clinicians should control additional factors such as host immunity. A previous study on fungal distribution inside and outside of two French hospitals demonstrated that fungal species in these two environments were markedly different [12]. However, epidemiology for environmental fungi is different according to the countries and centers. Since a previous Korean study was conducted on airborne microorganisms in hospital lobbies there have been no reports on the differences in the concentration and distribution of fungal species outside and within hospitals. In addition, the authors did not provide data for the spring season [10]. On the other hand, there are relatively small numbers of studies on microflora in cleanrooms. According to one review, it is known that cleanroom microflora are mostly composed of Gram-positive bacteria, and fungi represent about 1% [13]. Therefore, it is necessary to evaluate fungal epidemiology with seasonal variations both outside and within hospitals.

The aim of the present study was to evaluate the concentration and distribution of fungal species identified in air collected from both outside and inside in three different locations in a hematology unit (with and without access to HEPA filters) of a hospital in Korea over the course of one year.

## 2. Methods

### 2.1. Hospital Setting, Location and Study Design

One year of prospective air sampling was conducted (every two weeks from 24 May 2017 to May 2018) at Seoul St. Mary’s Hospital, a tertiary referral medical center located in Seoul, South Korea (37°50.039′ N, 127°00.665′ W). Seoul St. Mary’s Hospital is the biggest medical center for HSCT in Asia, performing over 500 HSCTs annually. A HVAC system was installed therein to clean the air inside the hospital building. In addition, a positive-pressure HEPA filter system was installed in the wards for chemotherapy and HSCT, with an ISO (International Organization for Standardization) 7-class cleanroom grade for hallways, and an ISO 5-class cleanroom grade for patients’ rooms [14]. The air sampling was performed at four separate locations: outside the building (O); in the general ward (GW) of the hematology unit on the 19th floor; in a lounge in the chemotherapy ward on the 19th floor (CR_L_, ISO 7-class for the cleanroom); and in a patient’ room in the chemotherapy ward on the 19th floor (CR_R_, ISO 5 class for the cleanroom).

### 2.2. Sampling Methods

An Andersen single-stage air sampler was placed 1.30 m from the ground outside the building, and 1.30 m from the floor of the room or lounge. Three consecutive air samplings were performed in one location at 20-min intervals. A KAS-110 apparatus (KEMIK Cooperation, Seongnam City, South Korea) was used to aspirate ambient air. Calibration was performed before air sampling using a KMF-200 airflow calibrator (KEMIK Cooperation). The sampler interior and cover were cleaned using a cotton ball soaked with a 70% ethanol solution. A petri dish containing Sabouraud dextrose agar (SDA) was then placed in the sampler. The air sampler passed air at a rate of 16 L/min for 15 min. The total volume collected was 240 L in each sample. The impactor (74 mm height × 105 mm diameter) had a pattern of 400 calibrated holes (0.25 mm diameter). The samplings were performed simultaneously (between 14:00 and 15:30 h) at four different locations (O, GW, CR_L_, and CR_R_) in consideration of daily variation. A total of 288 SDA plates were collected from three consecutive collections at each of the four designated locations every two weeks for 12 months. The temperature and relative humidity were determined at the start of sampling.

### 2.3. Colony Counting Methods

To optimize culture and isolation of various environmental fungi, the SDA plates were incubated at 35 °C for 96 h, and monitored daily. Fungal colonies were counted and distinguished from bacterial colonies on the basis of morphology and inverted microscopic feature. Observed fungal colonies and adjusted CFU, according to the Anderson sampler positive-hole conversion table [15], and concentrations for the sampled volume (m^3^) (CFU/m^3^) were calculated. The data are presented as GMs.

### 2.4. Fungal Identification

Fungal isolates were sub-cultured on SDA agar at 35 °C, and identified on the basis of macroscopic and microscopic morphology. The conidia were harvested in phosphate-buffered saline with 0.01% Tween 80, and washed with ultrapure water. Fungal DNA was extracted using MasterPure^TM^ DNA purification kit (Epicentre, Madison, WI, USA) following the manufacturer’s instructions. Sequence-based analysis of pan-fungal PCR assays targeting the internal transcribed spacer (ITS)-1 and -2 regions (ITS1–5.8S–ITS2) was used to detect all fungal species. The ITS1–5.8S–ITS2 region was amplified using ITS1-F KYO2 (5′-TAGAGGAAGTAAAAGTCGTAA-3′) and ITS4 (5′-TCCTCCGCTTATTGATATG-3′) primers, as previously described [16]. Briefly, the cycling conditions were as follows: 95 °C for 5 min; 30 cycles of 94 °C for 30 s, 50 °C for 30 s, and 72 °C for 1 min, followed by a final extension at 72 °C for 7 min.

### 2.5. Statistical Analysis

All statistical analyses were performed using SAS 9.4 software (SAS Institute, Cary, NC, USA). Chi-squared analysis was performed to evaluate the distribution of cultured fungal species within and outside the hospital. Analysis of variance (ANOVA) was performed to analyze the differences of CFU/m^3^ for different sites (O vs. GW vs. CR_R_). For seasonal comparison, the CFU/m^3^ was log-transformed. Duncan’s multiple range test was used for post hoc analysis. A *p* for trend between humid and log transformed fungal counts was derived from regression analysis; *p*-values smaller than 0.05 were considered as statistically significant.

## 3. Results

**Figure 1:**
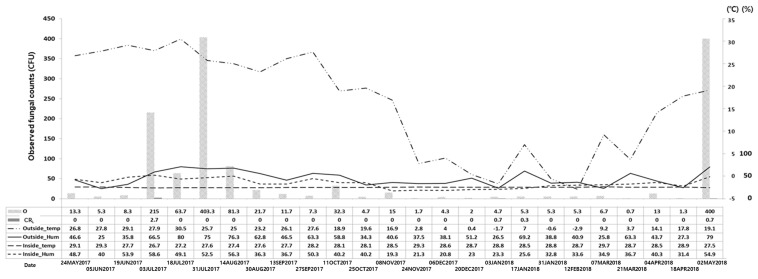
Trends in fungal density according to temperature and relative humidity. Abbreviations: CFU, colony-forming unit; CR_L_, lounge of the cleanroom; Hum, relative humidity; O, outside the hospital; Temp, temperature.

The mean temperature and relative humidity at each location are specified in Table 1 and Figure 1. The mean temperatures for the O and in the GW were 15.9 °C (SD: 11.3 °C; minimum [min]: −2.9 °C; maximum [max]: 30.5 °C) and 26.7 °C (SD: 0.9 °C; min: 24.7 °C; max: 29.0 °C), respectively. The mean relative humidity values for the O and in the GW ere 50.1% (SD: 17.9%; min: 25.0%; max: 80.0%) and 41.3% (SD: 12.6%; min: 22.4%; max: 64.0 °C), respectively.

The fungal burden in the air at each location (indoor and outdoor) is specified in Table 2, presented as crude fungal counts, adjusted counts, and fungal concentrations. Average of fungal concentration in outside was 954.8 CFU/m^3^ (range: 4.2–10,112.5 CFU/m^3^). However, fungal concentration in CR_R_ was not detected.

**Figure 2:**
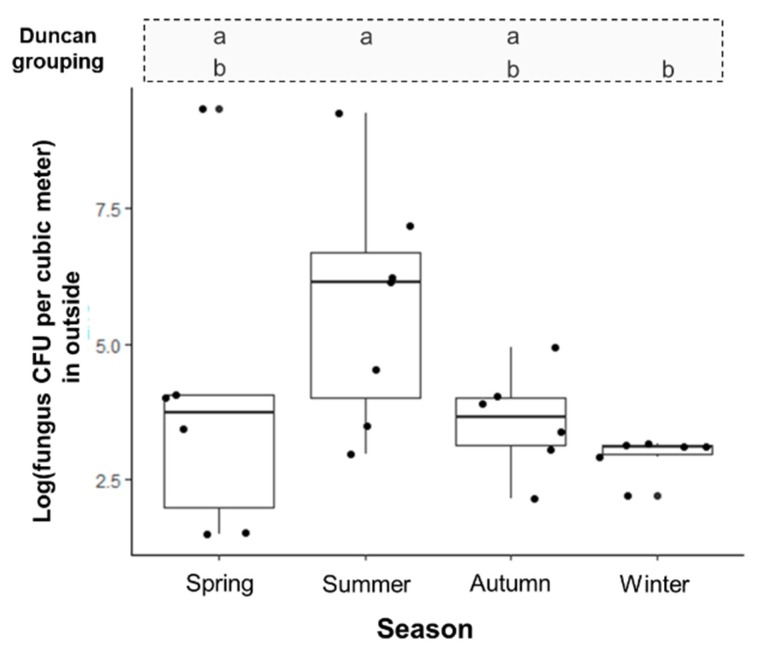
Seasonal variability of log fungal (CFU/m^3^) in the outside of hospital. Abbreviations: a, b: same group symbols by Duncan’s *post hoc* analysis, respectively.

The log transformed fungal count in the summer season was higher than that in any other season by ANOVA (*p* < 0.05) shows the annual variation of fungal concentration (Figure 2). After adjustment of both temperature and relative humidity together (R^2^ = 0.546), as relative humidity increased at outside, the outdoor fungal colony count also showed an increasing trend (beta: 0.078, *p* < 0.05) in multiple regression analysis. However, a statistical correlation with temperature was not observed.

**Figure 3:**
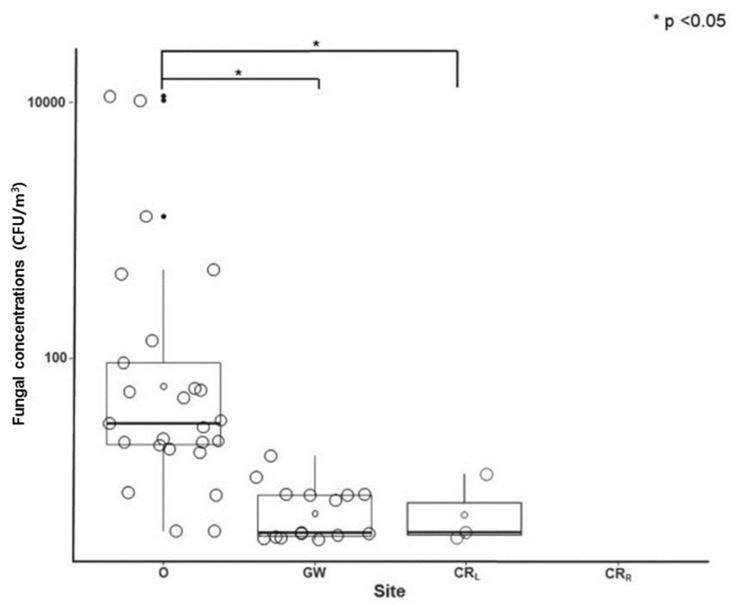
Comparison of fungal concentration by site. Abbreviations: CFU, colony-forming unit; CR_L_, lounge of the cleanroom; CR_R_, patients’ room in the cleanroom; GW, general wards; O, outside the hospital.

The comparison of fungal concentration (CFU/m^3^) by site is shown in Figure 3. The mean fungal density at outside of the hospital building was statistically higher than in all three locations within the hospital (GW vs. O, and CR_L_ vs. O) (*p* < 0.05). The calculated fungal concentration in CR_R_ was zero, and therefore no statistical analyses were performed.

**Figure 4:**
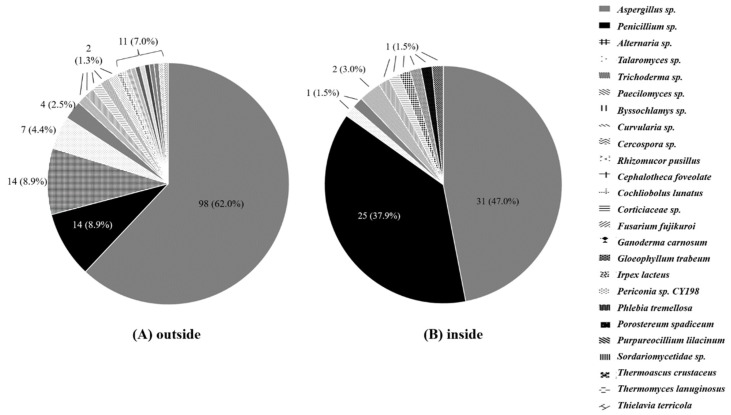
Fungal diversity within and outside the hospital building.

The results of species-level identification of the isolated fungi are summarized in Figure 4. *Aspergillus* was the most prevalent species both inside (47.0%) and outside (62.0%) of the hospital. However, the distribution of other species was different. In the outdoors, various molds, including *Aspergillus*, *Penicillium*, *Alternaria*, *Talaromyces*, *Trichoderma*, *Paecilomyces*, *Byssochlamys*, *Curvularia*, and *Cercospora*, were identified. *Penicillium* and *Alternaria* accounted for the second common species (8.9%), and *Talaromyces* was the fourth most common species (4.4%). Those *Talaromyces* species were all non-*marneffei Talaromyces*. On the other hand, within the hospital, *Penicillium* was the second most dominant fungi, accounting for 37.9% (*n* = 25), as compared to 8.9% (*n* = 14) found outside (*p* < 0.001). In overall, the third most common molds were *Alternaria* species.

The proportion of *Alternaria* species was found to significantly differ within (0%) and outside (8.9%) the hospital (*p* < 0.001). Although environmental fungi were identified from air in the hospital, all were isolated from the general ward, not from the HEPA-filtered cleanroom (*n* = 0).

The results of major top four species identified in the isolated fungi from the outside of hospital (by season) are summarized in Table 3. *Aspergillus* spp. was dominant outside the hospital in the summer, autumn, and winter seasons; however, *Alternaria* spp. was dominant in the spring season.

## 4. Discussion

In the present study, the concentration and distribution of fungi in the air were monitored within and outside of the hospital over one year. The distribution of mold was quite different between indoor and outdoor environment. Fungi isolated from the air collected outside of the hospital varied in both species and concentration depending on temperature and relative humidity. However, the fungi from indoor air of the hospital did not. The most common isolated fungal species was *Aspergillus*, followed by *Penicillium* both inside and outside of the hospital.

Hospitals in Korea should have equipment such as HVAC systems to maintain standard indoor air quality [17]. A previous study on the levels of airborne microorganisms in six hospital lobbies in Korea reported that the GM of fungal level was 59 CFU/m^3^ (SD: 2.2; range: 110–220 CFU/m^3^) for fungi [10]. Also, the daily peak fungal concentration was shown between 16:00 and 18:00, and the peak value was over 150 CFU/m^3^. These observations reflect the notion that the HVAC system may not be sufficient to reduce fungal exposure, even if those systems are working in the hospital lobbies throughout the day. The semi-open nature of the lobby can hinder the performance of the HVAC system, which might affect its efficiency. A relatively higher fungal count (85.9 CFU/m^3^) was reported in a Mexican hospital study [9]. However, according to the majority of previous studies, fungal exposure in a hospital environment equipped with a HEPA filter, such as a hematology unit, might be lower than that in HEPA-lacking environments (<5 CFU/m^3^) [5,6,7,8]. It is concordant with previous results that the mean fungal count inside the hospital was 26.5 CFU/m^3^ and the mean fungal counts in the cleanroom and lounges was below 5 CFU/m^3^ in the present study. There might be a difference between lobby and general wards/cleanroom environments in Korean studies. Therefore, an HVAC system and a positive-pressure system with a HEPA filter might effectively reduce fungal exposure via air within a hospital [18].

Movement of people and the associated airflow would induce an influx of fungal spores from the outside. In the present study, the fungal levels decreased as follows: O (954.8 CFU/m^3^) > GW (4.2 CFU/m^3^) > CR_L_ (0.7 CFU/m^3^) > CR_R_ (0 CFU/m^3^). The fungal concentration was greatly reduced between O and GW, which is considered to be possible given that the average fungal concentration in hospital lobbies was 59 CFU/m^3^ in another Korean study [10]. The hematology GW evaluated in this study is located on the 19th floor. The long air passage through the elevator and the HVAC system might lead to a reduction of fungal levels. However, the distribution of fungal species was different according to the location, as shown in Table 3. Among the total 224 environmental mold isolates, *Aspergillus* (57.6%, *n* = 129) species were the most dominant, followed by *Penicillium* (17.4%, *n* = 39). A relatively higher proportion of *Penicillium* species inside of the hospital observed in this study could reflect the possibility of the existence of another fungal source in the hospital, or the possibility of that it could originate from the outside. Fourteen *Penicillium* species samples from the outdoors were identified, 13 as *P. oxalicum* and one *P. citrinum.* On the other hand, in the indoor *Penicillium* samples the species were *P. hispanicum* (12), *P. citrinum* (6), and *P. chermesinum*, ect. (3), but *P. oxalicum* was not found. Those *Penicillium* isolates were all identified in both indoor and outdoor air (except in the spring season). This similarity suggests the possibility of environmental factors rather than the effects of the HVAC system. The non-*marneffei Penicillium* species were generally non-pathogenic fungi. From the perspective of nosocomial fungal infections, further study to determine the origin of fungi and clinical significance may be needed.

Relative humidity and temperature usually affect the fungal density outside the hospital building. Seasonal variation also affects fungal levels. As shown in Figure 1, fungal concentrations outside of the hospital in the spring and summer seasons were higher than in other seasons in this study. However, Park et al. reported that the fungal burden was higher in the autumn than in summer and winter [10]. Multiple regression analysis revealed a regression coefficient below 1 after adjusting for relative humidity and temperature (*p* < 0.05) [10]. This is different from the observations of the current study. Some differences in relative humidity and temperature between the present and previous studies are possible. The authors did not evaluate fungal concentrations in the spring, when the fungal concentrations were the highest (2 May 2018, when the humidity was 79%) in the present study. In addition, fungal levels outside the hospital were not determined. These differences might account for the discrepancies in the reported seasonal variation of fungal levels among different studies in Korea.

In this study, 21 and 11 fungal genera were identified from outside and inside the hospital, respectively. The higher diversity of fungi outside may reflect microbial community in the atmosphere [18]. The fungal distribution may also differ depending on the region. Sautour et al. reported that *Cladosporium* spp. was the dominant fungus outside a hospital in France. A study of the outdoor environment of one university in Minneapolis (MN, USA) reported that 94% of samples were positive for *Aspergillus* spp. [8]. The temperature and dew point determine the spore types in the outdoor environment. Another study correlating spore types in the spring with the environmental conditions in Tulsa (OK, USA) concluded that *Cladosporium* was dominant in a year characterized by reduced precipitation [19]. On the other hand, another report of cleanroom fungi concluded that the most common fungi in cleanrooms were *Aspergillus*, *Penicillium*, and *Trychophyton* [20]. In the present study, *Aspergillus* species were the dominant fungi outside of hospital in summer, autumn, and winter season; *Alternaria* species were dominant in the spring season. This result reflects that most dominant fungus was shown as *Aspergillus* spp., with seasonal variation. Figure 2 shows absolute fungal counts were seasonal; it seems that *Aspergillus* spp. might be related with high temperatures and relative humidity, as shown in Minneapolis over the summer through to the winter season [8]. However, a relationship between fungal microenvironment and any other related factors was not shown in the present study due to study was not designed for evaluating this. Therefore, data on dominant fungi should be interpreted in the light of the potential environmental factors (i.e., temperature, relative humidity, microenvironment, etc.) where the study was conducted.

The present study has several limitations. The first limitation is a potential selection bias. Fungal sampling was performed at only one hospital. In addition, sampling in the GW was performed in one site on the 19th floor, where a hematology unit was located. Nevertheless, the area was appropriate in order to make comparisons within different locations in the hematology unit, including a cleanroom equipped with a HEPA filter and a positive-pressure system. Second, the trend in changing fungal levels (O > GW > CR_L_ > CR_R_) was not validated by analyzing the lobby, which could be another limitation of the present study. Finally, daily variation in the concentration of airborne fungi [10] was not examined.

Regardless of the limitations, the current study has several strengths. First, the sampling was performed simultaneously (between 14:00 and 15:30 h) every other week throughout the year. Therefore, the seasonal variation could be determined in detail. Second, the indoor sampling sites were classified as being equipped with HVAC system (GW of the hematology unit), or with both the HVAC system and a positive-pressure HEPA filtered system (CR_L_ and CR_R_). Third, sampling at different sites was performed simultaneously.

In summary, *Aspergillus* sp. and *Penicillum* sp. are the most common species both within and outside the hospital. Fungal density affected by relative humidity, presenting higher mean fungal density in summer. The concentrations of fungi were found to be greatest outside, then in the GW, and finally in the cleanroom. In addition, the outdoor fungal profile was more diverse than the indoor profile.

In conclusion, the outdoor fungal profile is diverse even in Seoul, a highly urbanized area in Korea. The distribution of indoor air fungi is significantly different from outdoor due to the air quality system of hospital. Consequently, HVAC and HEPA filtering systems should be used to effectively reduce fungal counts in hospitals, to prevent potential nosocomial fungal infections.

## Figures and Tables

**Table 1:** Temperature and relative humidity profile at each location and by season.

	O			GW			CR_L_			CR_R_		
	Mean	SD	(Min., Max.)	Mean	SD	(Min., Max.)	Mean	SD	(Min., Max.)	Mean	SD	(Min., Max.)
Temperature (°C)	15.9	11.3	(−2.9, 30.5) *	26.7	0.9	(24.7, 29.0)	26.5	1.6	(23.5, 29.0)	28.3	0.7	(26.0, 29.0)
Spring	15.1	8.1	(3.7, 26.8)	26.3	0.8	(24.8, 27.0)	27.0	2.0	(24.1, 29.4)	28.7	0.7	(27.5, 29.7)
Summer	27.0	2.5	(23.2, 30.5)	27.2	1.1	(26.0, 29.0)	27.1	1.3	(25.8, 29.2)	27.6	0.8	(26.7, 29.3)
Autumn	18.7	8.8	(2.8, 27.6)	26.9	0.5	(26.2, 27.5)	26.0	1.2	(24.2, 27.4)	28.3	0.5	(27.7, 29.3)
Winter	1.0	3.8	(−2.9, 7.0)	26.4	1.0	(24.7, 27.5)	25.6	1.9	(23.5, 27.7)	28.7	0.1	(28.5, 28.8)
Relative humidity (%)	50.1	17.9	(25.0, 80.0) *	41.3	12.6	(22.4, 64.0)	40.0	11.3	(26.4, 62.1)	38.4	12.1	(19.3, 58.6)
Spring	47.6	20.7	(25.8, 79.0)	42.4	8.9	(33.6, 55.9)	38.6	10.1	(26.4, 52.0)	41.1	8.9	(31.4, 54.9)
Summer	60.2	21.4	(25.0, 80.0)	52.2	11.8	(38.9, 64.0)	51.0	9.0	(37.5, 62.1)	49.5	8.4	(36.3, 58.6)
Autumn	46.8	11.8	(34.3, 63.3)	37.7	13.5	(22.4, 56.8)	37.7	11.1	(27.4, 53.3)	34.7	12.0	(19.3, 50.3)
Winter	44.1	14.6	(26.5, 69.2)	31.2	5.0	(22.4, 37.3)	31.1	3.5	(27.0, 35.5)	26.5	5.4	(20.8, 33.6)

* *p* < 0.05 by ANOVA for temperature and relative humidity by location. Abbreviations: CR_L_, lounge of the cleanroom; CR_R_, patients’ room in the cleanroom; GW, general ward; Max., maximum; Min., minimum; O, outside the hospital; SD, standard deviation.

**Table 2:** Fungal colony count and concentrations at each location according to air sampling time throughout the year.

Date	Observed Fungal Count (CFU)				Adjusted Fungal Count (CFU)				Fungal Concentration (CFU/m^3^)			
	O	GW	CR_L_	CR_R_	O	GW	CR_L_	CR_R_	O	GW	CR_L_	CR_R_
24 May 2017	13.3	0.0	0	0	13	0	0	0	54.2	0.0	0.0	0
5 June 2017	5.3	0.0	0	0	5	0	0	0	20.8	0.0	0.0	0
19 June 2017	8.3	1.3	0	0	8	1	0	0	33.3	4.2	0.0	0
3 July 2017	286.5	1.3	2.7	0	505	1	2.7	0	2104.2	4.2	11.3	0
18 July 2017	94.0	2.0	0	0.3	107	2	0	0	445.8	8.3	0.0	0
31 July 2017	400.0	2.3	0	0.3	2427	2	0	0	10,112.5	8.3	0.0	0
14 August 2017	81.3	2.0	0	0	91	2	0	0	379.2	8.3	0.0	0
30 August 2017	21.7	4.0	0	0	22	4	0	0	91.7	16.7	0.0	0
13 September 2017	13.7	2.0	0	0	14	2	0	0	58.3	8.3	0.0	0
27 September 2017	7.3	2.7	0	0	7	3	0	0	29.2	12.5	0.0	0
11 October 2017	32.3	0.0	0	0	33	0	0	0	137.5	0.0	0.0	0
25 October 2017	4.7	0.3	0	0	5	0	0	0	20.8	0.0	0.0	0
8 November 2017	15.0	0.0	0	0	15	0	0	0	62.5	0.0	0.0	0
24 November 2017	1.7	1.0	0	0	2	1	0	0	8.3	4.2	0.0	0
6 December 2017	4.3	0.3	0	0	4	0	0	0	16.7	0.0	0.0	0
20 December 2017	2.0	1.0	0	0	2	1	0	0	8.3	4.2	0.0	0
3 January 2018	4.7	1.3	0.7	0.3	5	1	0.7	0	20.8	4.2	2.9	0
17 January 2018	5.3	0.3	0.3	0	5	0	0.3	0	20.8	0.0	1.3	0
31 January 2018	5.3	1.0	0	0	5	1	0	0	20.8	4.2	0.0	0
12 February 2018	5.3	1.7	0	0	5	2	0	0	20.8	8.3	0.0	0
7 March 2018	6.7	1.0	0	0	7	1	0	0	29.2	4.2	0.0	0
21 March 2018	0.7	0.3	0	0	1	0	0	0	4.2	0.0	0.0	0
4 April 2018	13.0	0.0	0	0.3	13	0	0	0	54.2	0.0	0.0	0
18 April 2018	1.3	0.0	0	0	1	0	0	0	4.2	0.0	0.0	0
2 May 2018	400.0	0.7	0.7	0	2427	1	0.7	0	10,112.5	4.2	2.9	0
Total (/Year)	1433.7	26.5	4.4	1.2	5729	25	4.4	0	23,870.8	104.3	0.7	0
Average (/sample)	57.35	1.06	0.18	0.05	229.2	1	0.18	0	954.8	4.1	0.7	0
Range	0.7–400	0–4.0	0–2.7	0–0.3	1–2427	0–4	0–2.7	0–0	4.2–10,112.5	0–16.7	0–11.3	0–0

Abbreviations: CFU, colony-forming unit; CR_L_, lounge of the cleanroom; CR_R_, patients’ room in the cleanroom; GW, general ward; O, outside the hospital.

**Table 3:** Seasonal distribution of four major fungus species in the outside of hospital throughout the year.

	Season				*p* Value
	Spring	Summer	Autumn	Winter	
*Aspergillus* sp.	5 (33.3)	27 (77.1)	38 (84.4)	28 (73.7)	<0.001
*Penicillium* sp.	0 (0.0)	4 (11.4)	6 (13.3)	4 (10.5)	
*Alternaria* sp.	10 (66.7)	2 (5.7)	0 (0.0)	2 (5.3)	
*Talaromyces* sp.	0 (0.0)	2 (5.7)	1 (2.2)	4 (10.5)	

## References

[1] Shelton B.G., Kirkland K.H., Flanders W.D., Morris G.K. (2002). Profiles of airborne fungi in buildings and outdoor environments in the United States. Appl. Environ. Microbiol..

[2] Gangneux J.P., Bougnoux M.E., Hennequin C., Godet C., Chandenier J., Denning D.W., Dupont B. (2016). An estimation of burden of serious fungal infections in France. J. Mycol. Med..

[3] Ullmann A.J., Aguado J.M., Arikan-Akdagli S., Denning D.W., Groll A.H., Lagrou K., Lass-Flörl C., Lewis R.E., Munoz P., Verweij P.E. (2018). Diagnosis and management of *Aspergillus* diseases: Executive summary of the 2017 ESCMID-ECMM-ERS guideline. Clin. Microbiol. Infect..

[4] Roemer T., Krysan D.J. (2014). Antifungal Drug Development: Challenges, Unmet Clinical Needs, and New Approaches. Cold Spring Harb. Perspect. Med..

[5] Bellanger A.P., Reboux G., Demonmerot F., Gbaguidi-Haore H., Millon L. (2017). Fungal aerocontamination exposure risk for patients in 3 successive locations of a pediatric hematology unit department: Influence of air equipment and building structure on air quality. Am. J. Infect. Control.

[6] Heutte N., Andre V., Dubos Arvis C., Bouchart V., Lemarie F., Legendre P., Votier E., Louis M.Y., Madelaine S., Seguin V. (2017). Assessment of multi-contaminant exposure in a cancer treatment center: A 2-year monitoring of molds, mycotoxins, endotoxins, and glucans in bioaerosols. Environ. Monit. Assess..

[7] Araujo R., Carneiro A., Costa-Oliveira S., Pina-Vaz C., Rodrigues A.G., Guimaraes J.E. (2008). Fungal infections after haematology unit renovation: Evidence of clinical, environmental and economical impact. Eur. J. Haematol..

[8] Falvey D.G., Streifel A.J. (2007). Ten-year air sample analysis of *Aspergillus* prevalence in a university hospital. J. Hosp. Infect..

[9] Martinez-Herrera E.O., Frias De-Leon M.G., Duarte-Escalante E., Calderon-Ezquerro Mdel C., Jimenez-Martinez Mdel C., Acosta-Altamirano G., Rivera-Becerril F., Toriello C., Reyes Montes Mdel R. (2016). Fungal diversity and *Aspergillus* species in hospital environments. Ann. Agric. Environ. Med..

[10] Park D.U., Yeom J.K., Lee W.J., Lee K.M. (2013). Assessment of the levels of airborne bacteria, Gram-negative bacteria, and fungi in hospital lobbies. Int. J. Environ. Res. Public Health.

[11] Yahara K., Miura M., Masunaga K., Matsumoto K., Miyao T., Tanamachi C., Hashimoto K., Sagawa K., Watanabe H. (2010). Comparison of two control measures of weatherstripping in reducing blowing dust during hospital renovations. J. Infect. Chemother. Off. J. Jpn. Soc. Chemother..

[12] Sautour M., Sixt N., Dalle F., L’Ollivier C., Fourquenet V., Calinon C., Paul K., Valvin S., Maurel A., Aho S. (2009). Profiles and seasonal distribution of airborne fungi in indoor and outdoor environments at a French hospital. Sci. Total Environ..

[13] Sandle T. (2011). A review of cleanroom microflora: Types, trends, and patterns. PDA J. Pharm. Sci. Technol..

[14] International Organization for Standardization (ISO) (2015). Cleanrooms and Associated Controlled Environments—Part 1: Classification of Air Cleanliness by Particle Concentration.

[15] Andersen A.A. (1958). New sampler for the collection, sizing, and enumeration of viable airborne particles. J. Bacteriol..

[16] Toju H., Tanabe A.S., Yamamoto S., Sato H. (2012). High-coverage ITS primers for the DNA-based identification of ascomycetes and basidiomycetes in environmental samples. PLoS ONE.

[17] Department of Environment (2018). Enforcement Decree of Indoor Air Quality Control in Public-Use Facilities, etc. Act.

[18] Woo C., An C., Xu S., Yi S.M., Yamamoto N. (2018). Taxonomic diversity of fungi deposited from the atmosphere. ISME J..

[19] Ortiz G., Yague G., Segovia M., Catalan V. (2009). A study of air microbe levels in different areas of a hospital. Curr. Microbiol..

[20] Troutt C., Levetin E. (2001). Correlation of spring spore concentrations and meteorological conditions in Tulsa, Oklahoma. Int. J. Biometeorol..

